# *N*-(2-Hy­droxy-1,1-dimethyl­eth­yl)­benzene­sulfonamide

**DOI:** 10.1107/S1600536810044144

**Published:** 2010-10-31

**Authors:** Aziz-ur-Rehman, Nadia Abbas, Mehmet Akkurt, Muhammad Athar Abbasi, Shahzad Sharif, Islam Ullah Khan

**Affiliations:** a Department of Chemistry, Government College University, Lahore 54000, Pakistan; b Department of Physics, Faculty of Arts and Sciences, Erciyes University, 38039 Kayseri, Turkey

## Abstract

In the title mol­ecule, C_10_H_15_NO_3_S, the S atom is bonded in a distorted tetra­hedral geometry. In the crystal structure, inter­molecular N—H⋯O, O—H⋯O and weak C—H⋯O hydrogen bonds connect the mol­ecules to form a two-dimensional network parallel to (100). The 2-methyl­propan-1-ol group is disordered over two orientations with occupancies of 0.570 (3) and 0.430 (3).

## Related literature

For general background to sulfonamide derivatives, see: Ozbek *et al.* (2007 ▶); Parari *et al.* (2008 ▶). For our previous structural studies on sulfonamide derivatives, see: Asiri *et al.* (2009 ▶); Aziz-ur-Rehman *et al.* (2010 ▶).
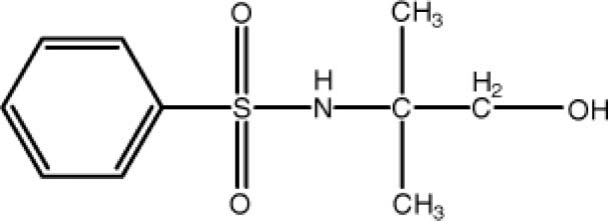


## Experimental

### 

#### Crystal data

C_10_H_15_NO_3_S
*M* *_r_* = 229.30
Monoclinic, 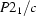

*a* = 12.4094 (3) Å
*b* = 9.0042 (2) Å
*c* = 10.4525 (2) Å
β = 93.731 (1)°
*V* = 1165.45 (4) Å^3^
*Z* = 4
Mo *K*α radiation
μ = 0.27 mm^−1^
*T* = 296 K
0.24 × 0.16 × 0.07 mm

#### Data collection

Bruker APEXII CCD diffractometer
10723 measured reflections
2857 independent reflections
2331 reflections with *I* > 2σ(*I*)
*R*_int_ = 0.024

#### Refinement

*R*[*F* ^2^ > 2σ(*F* ^2^)] = 0.039
*wR*(*F* ^2^) = 0.119
*S* = 1.04
2857 reflections
176 parameters
8 restraints
H-atom parameters constrained
Δρ_max_ = 0.31 e Å^−3^
Δρ_min_ = −0.34 e Å^−3^

### 

Data collection: *APEX2* (Bruker, 2007 ▶); cell refinement: *SAINT* (Bruker, 2007 ▶); data reduction: *SAINT*; program(s) used to solve structure: *SHELXS97* (Sheldrick, 2008 ▶); program(s) used to refine structure: *SHELXL97* (Sheldrick, 2008 ▶); molecular graphics: *PLATON* (Spek, 2009 ▶); software used to prepare material for publication: *WinGX* (Farrugia, 1999 ▶) and *PLATON*.

## Supplementary Material

Crystal structure: contains datablocks global, I. DOI: 10.1107/S1600536810044144/lh5159sup1.cif

Structure factors: contains datablocks I. DOI: 10.1107/S1600536810044144/lh5159Isup2.hkl

Additional supplementary materials:  crystallographic information; 3D view; checkCIF report

## Figures and Tables

**Table 1:** Hydrogen-bond geometry (Å, °)

*D*—H⋯*A*	*D*—H	H⋯*A*	*D*⋯*A*	*D*—H⋯*A*
N1—H1*A*⋯O3*B*^i^	0.86	2.14	2.840 (9)	139
O3*B*—H3*B*⋯O2^ii^	0.82	2.02	2.777 (10)	152
C10*B*—H10*D*⋯O1^iii^	0.97	2.54	3.478 (3)	162

Symmetry codes: (i) 
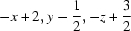
; (ii) 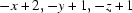; (iii) 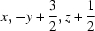.

**Fig. 1.**
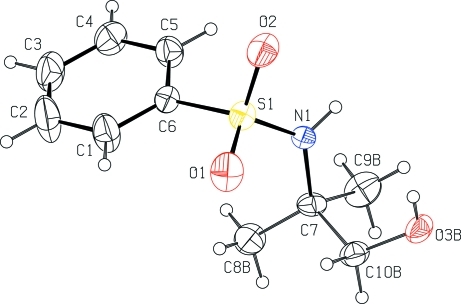
The molecular structure of (I) with displacement ellipsoids drawn at the 30% probability level. The disorder is not shown.

**Fig. 2.**
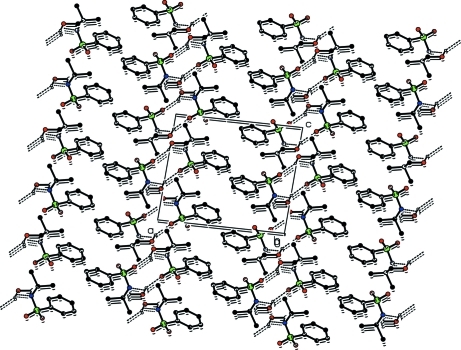
Part of the crystal structure of (I) H atoms not involved in hydrogen bonds (dashed lines) have been omitted for clarity and only the major component of the disorder is shown.

## supplementary crystallographic information

### Comment

The sulfonamide moiety is found in a number of synthetic as well as natural compounds and renders various biological acitivities in these compounds (Ozbek *et al.*, 2007, Parari *et al.*, 2008). In continuation of our structural studies on various sulfonamide derivatives (Asiri *et al.*, 2009, Aziz-ur-Rehman *et al.*, 2010) herein we report the crystal structure of the title compound (I).

The molecular structure of (I) is shown in Fig. 1 and reveals a distorted tetrahedral geometry around the S atom [maximum deviation from the ideal sp^3^ hybridized geometry for O—S—O = 119.29 (8)°]. There are weak intramolecular C—H···O contacts within the molecule. In the crystal structure, intermolecular N—H···O, O—H···O and weak C—H···O hydrogen bonds connect the molecules to form a two-dimensional network parallel to (100) (Fig. 2).

### Experimental

A mixture of benzenesulfonyl chloride (10.0 mmol; 1.28 ml), 2-amino-2-methyl propanol (10.0 mmol; 0.95 ml), aqueous sodium carbonate (10%; 15.0 ml) and water (20 ml) was stirred for one and half hours at room temperature. The crude mixture was washed with distilled water and dried. The product was dissolved in methanol and recrystallized by slow evaporation of the solvent, to generate colourless crystals of *N*-(2-hydroxy-1,1-dimethylethyl)benzenesulfonamide in 65% yield.

### Refinement

All H atoms were positioned geometrically with N—H = 0.86, O—H = 0.82 and C—H = 0.93–0.97 Å and allowed to ride on their parent atoms, with *U*_iso_(H) = 1.2*U*_eq_(C, N) or 1.5*U*_eq_(C_methyl_,O). The 2-methylpropan-1-ol group of the title molecule show disoder over two sets of sites with an occupancy ratio of 0.570 (3):0.430 (3).

### Crystal data

**Table:**

C_10_H_15_NO_3_S	*F*(000) = 488
*M**_r_* = 229.30	*D*_x_ = 1.307 Mg m^−^^3^
Monoclinic, *P*2_1_/*c*	Mo *K*α radiation, λ = 0.71073 Å
Hall symbol: -P 2ybc	Cell parameters from 5041 reflections
*a* = 12.4094 (3) Å	θ = 2.8–28.2°
*b* = 9.0042 (2) Å	µ = 0.27 mm^−^^1^
*c* = 10.4525 (2) Å	*T* = 296 K
β = 93.731 (1)°	Block, colourless
*V* = 1165.45 (4) Å^3^	0.24 × 0.16 × 0.07 mm
*Z* = 4	

### Data collection

**Table:**

Bruker APEXII CCD diffractometer	2331 reflections with *I* > 2σ(*I*)
Radiation source: sealed tube	*R*_int_ = 0.024
graphite	θ_max_ = 28.3°, θ_min_ = 3.4°
φ and ω scans	*h* = −16→16
10723 measured reflections	*k* = −12→10
2857 independent reflections	*l* = −13→11

### Refinement

**Table:**

Refinement on *F*^2^	Primary atom site location: structure-invariant direct methods
Least-squares matrix: full	Secondary atom site location: difference Fourier map
*R*[*F*^2^ > 2σ(*F*^2^)] = 0.039	Hydrogen site location: inferred from neighbouring sites
*wR*(*F*^2^) = 0.119	H-atom parameters constrained
*S* = 1.04	*w* = 1/[σ^2^(*F*_o_^2^) + (0.0674*P*)^2^ + 0.1878*P*] where *P* = (*F*_o_^2^ + 2*F*_c_^2^)/3
2857 reflections	(Δ/σ)_max_ = 0.001
176 parameters	Δρ_max_ = 0.31 e Å^−^^3^
8 restraints	Δρ_min_ = −0.33 e Å^−^^3^

### Special details

**Table:**

Geometry. Bond distances, angles *etc*. have been calculated using the rounded fractional coordinates. All su's are estimated from the variances of the (full) variance-covariance matrix. The cell e.s.d.'s are taken into account in the estimation of distances, angles and torsion angles
Refinement. Refinement on *F*^2^ for ALL reflections except those flagged by the user for potential systematic errors. Weighted *R*-factors *wR* and all goodnesses of fit *S* are based on *F*^2^, conventional *R*-factors *R* are based on *F*, with *F* set to zero for negative *F*^2^. The observed criterion of *F*^2^ > σ(*F*^2^) is used only for calculating -*R*-factor-obs *etc*. and is not relevant to the choice of reflections for refinement. *R*-factors based on *F*^2^ are statistically about twice as large as those based on *F*, and *R*-factors based on ALL data will be even larger.

### Fractional atomic coordinates and isotropic or equivalent isotropic displacement parameters (Å^2^)

**Table:**

	*x*	*y*	*z*	*U*_iso_*/*U*_eq_	Occ. (<1)
S1	0.81238 (3)	0.43637 (4)	0.53924 (3)	0.0453 (1)	
O1	0.76229 (12)	0.56081 (15)	0.47567 (14)	0.0735 (5)	
O2	0.88776 (11)	0.34876 (16)	0.47376 (13)	0.0715 (5)	
O3B	0.9863 (5)	0.7719 (10)	0.7102 (11)	0.0594 (14)	0.570 (3)
N1	0.87618 (9)	0.48927 (13)	0.66870 (11)	0.0397 (3)	
C1	0.60374 (15)	0.3406 (3)	0.5338 (2)	0.0764 (7)	
C2	0.52364 (17)	0.2428 (3)	0.5643 (3)	0.1035 (10)	
C3	0.54859 (17)	0.1204 (3)	0.6369 (3)	0.0838 (8)	
C4	0.65240 (17)	0.0938 (2)	0.6815 (2)	0.0694 (7)	
C5	0.73310 (14)	0.1902 (2)	0.65250 (18)	0.0581 (5)	
C6	0.70811 (11)	0.31395 (16)	0.57831 (14)	0.0437 (4)	
C7	0.83869 (12)	0.59545 (16)	0.76557 (14)	0.0444 (4)	
C8B	0.7250 (3)	0.5962 (4)	0.7901 (4)	0.0746 (13)	0.570 (3)
C9B	0.9096 (4)	0.5645 (4)	0.8934 (3)	0.0733 (13)	0.570 (3)
C10B	0.8755 (2)	0.7543 (3)	0.7249 (3)	0.0519 (9)	0.570 (3)
O3A	0.9831 (10)	0.7515 (14)	0.7318 (14)	0.085 (3)	0.430 (3)
C8A	0.7815 (4)	0.4982 (5)	0.8656 (4)	0.0567 (11)	0.430 (3)
C9A	0.7497 (4)	0.7001 (5)	0.7058 (4)	0.0597 (12)	0.430 (3)
C10A	0.9310 (3)	0.6663 (4)	0.8205 (3)	0.0494 (11)	0.430 (3)
H3B	1.00300	0.72570	0.64690	0.0890*	0.570 (3)
H4	0.66860	0.01040	0.73160	0.0830*	
H5	0.80410	0.17220	0.68260	0.0700*	
H8B1	0.68280	0.61690	0.71190	0.1120*	0.570 (3)
H8B2	0.71190	0.67140	0.85230	0.1120*	0.570 (3)
H8B3	0.70510	0.50100	0.82230	0.1120*	0.570 (3)
H9B1	0.98460	0.56600	0.87620	0.1100*	0.570 (3)
H10C	0.83670	0.77980	0.64430	0.0620*	0.570 (3)
H10D	0.85420	0.82490	0.78860	0.0620*	0.570 (3)
H9B2	0.89140	0.46890	0.92650	0.1100*	0.570 (3)
H9B3	0.89580	0.63970	0.95540	0.1100*	0.570 (3)
H1	0.58690	0.42360	0.48350	0.0920*	
H1A	0.93950	0.45220	0.68430	0.0480*	
H2	0.45240	0.26060	0.53530	0.1240*	
H3	0.49430	0.05440	0.65610	0.1010*	
H8A1	0.71950	0.45040	0.82410	0.0850*	0.430 (3)
H8A2	0.75910	0.56040	0.93360	0.0850*	0.430 (3)
H8A3	0.83100	0.42430	0.90030	0.0850*	0.430 (3)
H3A	0.99830	0.69890	0.67140	0.1280*	0.430 (3)
H9A1	0.68990	0.64200	0.67100	0.0890*	0.430 (3)
H9A2	0.77850	0.75770	0.63860	0.0890*	0.430 (3)
H9A3	0.72560	0.76540	0.77070	0.0890*	0.430 (3)
H10A	0.91060	0.72980	0.88990	0.0590*	0.430 (3)
H10B	0.98080	0.59190	0.85660	0.0590*	0.430 (3)

### Atomic displacement parameters (Å^2^)

**Table:**

	*U*^11^	*U*^22^	*U*^33^	*U*^12^	*U*^13^	*U*^23^
S1	0.0485 (2)	0.0503 (2)	0.0378 (2)	−0.0069 (2)	0.0083 (2)	0.0011 (1)
O1	0.0798 (9)	0.0732 (9)	0.0657 (8)	−0.0128 (7)	−0.0080 (7)	0.0318 (7)
O2	0.0709 (8)	0.0829 (9)	0.0644 (8)	−0.0190 (7)	0.0321 (6)	−0.0308 (7)
O3B	0.053 (2)	0.0512 (19)	0.077 (3)	−0.0173 (17)	0.0263 (17)	−0.0205 (18)
N1	0.0355 (6)	0.0386 (6)	0.0455 (6)	0.0017 (4)	0.0061 (5)	−0.0065 (5)
C1	0.0514 (9)	0.0801 (13)	0.0952 (15)	−0.0080 (9)	−0.0131 (9)	0.0279 (12)
C2	0.0492 (11)	0.116 (2)	0.142 (2)	−0.0234 (12)	−0.0186 (12)	0.0368 (19)
C3	0.0632 (12)	0.0812 (14)	0.1073 (17)	−0.0282 (11)	0.0084 (11)	0.0083 (13)
C4	0.0739 (12)	0.0561 (10)	0.0788 (13)	−0.0114 (9)	0.0095 (10)	0.0117 (9)
C5	0.0491 (8)	0.0577 (9)	0.0672 (10)	−0.0033 (7)	0.0012 (7)	0.0100 (8)
C6	0.0440 (7)	0.0453 (7)	0.0417 (7)	−0.0050 (6)	0.0026 (6)	−0.0020 (6)
C7	0.0521 (8)	0.0413 (7)	0.0414 (7)	0.0049 (6)	0.0152 (6)	−0.0027 (6)
C8B	0.0581 (18)	0.078 (2)	0.092 (3)	−0.0108 (17)	0.0389 (18)	−0.029 (2)
C9B	0.102 (3)	0.076 (2)	0.0411 (15)	−0.0045 (19)	−0.0006 (16)	−0.0051 (15)
C10B	0.0560 (17)	0.0421 (14)	0.0591 (16)	−0.0018 (12)	0.0151 (12)	−0.0096 (12)
O3A	0.100 (5)	0.080 (5)	0.080 (5)	−0.055 (4)	0.040 (3)	−0.042 (4)
C8A	0.070 (2)	0.053 (2)	0.0499 (19)	−0.0027 (18)	0.0259 (18)	0.0017 (17)
C9A	0.068 (2)	0.055 (2)	0.056 (2)	0.0252 (19)	0.0035 (18)	−0.0043 (17)
C10A	0.0512 (19)	0.056 (2)	0.0415 (18)	−0.0101 (15)	0.0059 (14)	−0.0122 (16)

### Geometric parameters (Å, °)

**Table:**

S1—O1	1.4247 (14)	C1—H1	0.9300
S1—O2	1.4315 (14)	C2—H2	0.9300
S1—N1	1.5954 (12)	C3—H3	0.9300
S1—C6	1.7677 (14)	C4—H4	0.9300
O3A—C10A	1.394 (14)	C5—H5	0.9300
O3B—C10B	1.402 (7)	C8A—H8A2	0.9600
O3A—H3A	0.8200	C8A—H8A1	0.9600
O3B—H3B	0.8200	C8A—H8A3	0.9600
N1—C7	1.4890 (19)	C8B—H8B1	0.9600
N1—H1A	0.8600	C8B—H8B2	0.9600
C1—C6	1.369 (2)	C8B—H8B3	0.9600
C1—C2	1.381 (3)	C9A—H9A3	0.9600
C2—C3	1.362 (4)	C9A—H9A1	0.9600
C3—C4	1.362 (3)	C9A—H9A2	0.9600
C4—C5	1.374 (3)	C9B—H9B1	0.9600
C5—C6	1.381 (2)	C9B—H9B2	0.9600
C7—C9A	1.552 (5)	C9B—H9B3	0.9600
C7—C10B	1.569 (3)	C10A—H10B	0.9700
C7—C8A	1.569 (5)	C10A—H10A	0.9700
C7—C8B	1.450 (4)	C10B—H10D	0.9700
C7—C10A	1.401 (4)	C10B—H10C	0.9700
C7—C9B	1.576 (4)		
			
S1···H8B1	2.9800	H8A1···C6	2.8400
S1···H9A1	2.8100	H1A···H3A	2.3400
S1···H9A2	3.1100	H1A···H9B1	2.2900
O1···C8B	3.364 (4)	H1A···H10B	2.2300
O1···C10B	3.364 (3)	H1A···O3B^v^	2.1400
O1···C9A	2.726 (4)	H1A···C10B^v^	3.0100
O2···O3B^i^	2.777 (10)	H1A···O3A^v^	2.2000
O2···O3A^i^	2.907 (14)	H1A···C10A^v^	3.0400
O3A···N1^ii^	2.912 (13)	H1A···O3A	2.7900
O3A···N1	2.767 (13)	H2···H8B2^vi^	2.5500
O3A···O2^i^	2.907 (14)	H8A2···H9A3	2.5300
O3B···N1	2.908 (9)	H8A2···H10A	2.4900
O3B···O2^i^	2.777 (10)	H3···C8B^vi^	2.8400
O3B···N1^ii^	2.840 (9)	H3···H8B3^vi^	2.5500
O3B···C9B^ii^	3.157 (10)	H8A3···H10B	2.4600
O1···H1	2.5100	H8A3···O2^vii^	2.6600
O1···H8B1	2.7600	H3A···N1	2.4200
O1···H9A3^iii^	2.6700	H3A···H1A	2.3400
O1···H4^iv^	2.8100	H3A···O2^i^	2.1800
O1···H8B2^iii^	2.7900	H3B···O2^i^	2.0200
O1···H10A^iii^	2.8200	H3B···N1	2.6700
O1···H9A1	2.4000	H4···C9A^xi^	2.9900
O1···H10C	2.7600	H4···H9A3^xi^	2.3400
O1···H10D^iii^	2.5400	H4···O1^vii^	2.8100
O1···H9A2	2.4600	H9A1···S1	2.8100
O2···H8A3^iv^	2.6600	H9A1···O1	2.4000
O2···H9B2^iv^	2.9000	H9A1···H8A1	2.3700
O2···H3A^i^	2.1800	H5···O3B^v^	2.9100
O2···H3B^i^	2.0200	H5···O3A^v^	2.8200
O3A···H5^ii^	2.8200	H9A2···S1	3.1100
O3A···H9A2	2.6600	H9A2···O1	2.4600
O3A···H1A^ii^	2.2000	H9A2···O3A	2.6600
O3A···H1A	2.7900	H9A3···H4^x^	2.3400
O3B···H9B1	2.5400	H9A3···H8A2	2.5300
O3B···H1A^ii^	2.1400	H9A3···O1^xii^	2.6700
O3B···H5^ii^	2.9100	H9A3···H10A	2.5600
O3B···H9B2^ii^	2.7900	H8B1···C6	3.0900
O3B···H9B1^ii^	2.8300	H8B1···H10C	2.5400
N1···O3A^v^	2.912 (13)	H8B1···S1	2.9800
N1···O3B	2.908 (9)	H8B1···O1	2.7600
N1···O3B^v^	2.840 (9)	H8B2···H9B3	2.4800
N1···O3A	2.767 (13)	H8B2···H10D	2.3700
N1···H3A	2.4200	H8B2···H2^viii^	2.5500
N1···H3B	2.6700	H8B2···O1^xii^	2.7900
C3···C8B^vi^	3.535 (4)	H8B3···C6	3.0600
C5···C8A	3.583 (5)	H8B3···H3^viii^	2.5500
C5···C8A^iv^	3.531 (5)	H8B3···H9B2	2.5100
C6···C8B	3.368 (4)	H9B1···H1A	2.2900
C6···C8A	3.499 (5)	H9B1···O3B	2.5400
C8A···C5^vii^	3.531 (5)	H9B1···H9B2^ix^	2.5100
C8A···C6	3.499 (5)	H9B1···O3B^v^	2.8300
C8A···C5	3.583 (5)	H9B1···C9B^ix^	2.9100
C8B···C6	3.368 (4)	H10A···H8A2	2.4900
C8B···O1	3.364 (4)	H10A···H9A3	2.5600
C8B···C3^viii^	3.535 (4)	H10A···O1^xii^	2.8200
C9A···O1	2.726 (4)	H10B···H1A	2.2300
C9B···O3B^v^	3.157 (10)	H10B···H8A3	2.4600
C9B···C9B^ix^	3.271 (6)	H10C···H9B3^iii^	2.2700
C10B···O1	3.364 (3)	H10C···O1	2.7600
C5···H8A1	2.9600	H10C···H8B1	2.5400
C6···H8B1	3.0900	H10D···O1^xii^	2.5400
C6···H8B3	3.0600	H10D···H8B2	2.3700
C6···H8A1	2.8400	H10D···H9B3	2.4400
C8B···H3^viii^	2.8400	H9B2···O3B^v^	2.7900
C9A···H4^x^	2.9900	H9B2···H8B3	2.5100
C9B···H9B2^ix^	3.0200	H9B2···C9B^ix^	3.0200
C9B···H9B1^ix^	2.9100	H9B2···H9B1^ix^	2.5100
C10A···H1A^ii^	3.0400	H9B2···O2^vii^	2.9000
C10B···H1A^ii^	3.0100	H9B3···H8B2	2.4800
C10B···H9B3^iii^	3.0000	H9B3···H10D	2.4400
H1···O1	2.5100	H9B3···C10B^xii^	3.0000
H8A1···C5	2.9600	H9B3···H10C^xii^	2.2700
H8A1···H9A1	2.3700		
			
O1—S1—O2	119.29 (8)	C5—C4—H4	120.00
O1—S1—N1	109.76 (7)	C3—C4—H4	120.00
O1—S1—C6	107.13 (8)	C4—C5—H5	120.00
O2—S1—N1	105.45 (7)	C6—C5—H5	120.00
O2—S1—C6	106.13 (8)	H8A1—C8A—H8A3	110.00
N1—S1—C6	108.70 (7)	H8A2—C8A—H8A3	109.00
C10A—O3A—H3A	109.00	C7—C8A—H8A1	109.00
C10B—O3B—H3B	109.00	C7—C8A—H8A2	109.00
S1—N1—C7	127.50 (10)	C7—C8A—H8A3	109.00
S1—N1—H1A	116.00	H8A1—C8A—H8A2	110.00
C7—N1—H1A	116.00	C7—C8B—H8B1	109.00
C2—C1—C6	119.3 (2)	C7—C8B—H8B2	109.00
C1—C2—C3	120.2 (2)	H8B1—C8B—H8B3	109.00
C2—C3—C4	120.6 (2)	C7—C8B—H8B3	109.00
C3—C4—C5	120.0 (2)	H8B1—C8B—H8B2	109.00
C4—C5—C6	119.56 (16)	H8B2—C8B—H8B3	110.00
S1—C6—C1	120.19 (14)	C7—C9A—H9A1	110.00
S1—C6—C5	119.46 (11)	H9A2—C9A—H9A3	110.00
C1—C6—C5	120.35 (16)	C7—C9A—H9A3	109.00
N1—C7—C10B	106.97 (15)	H9A1—C9A—H9A2	109.00
N1—C7—C10A	106.72 (18)	C7—C9A—H9A2	109.00
N1—C7—C8A	105.65 (19)	H9A1—C9A—H9A3	109.00
N1—C7—C9A	111.21 (19)	C7—C9B—H9B2	109.00
C9B—C7—C10B	103.6 (2)	C7—C9B—H9B3	109.00
C8A—C7—C9A	105.5 (3)	C7—C9B—H9B1	109.00
C8A—C7—C10A	112.0 (2)	H9B1—C9B—H9B3	110.00
C9A—C7—C10A	115.5 (2)	H9B2—C9B—H9B3	109.00
N1—C7—C8B	118.28 (19)	H9B1—C9B—H9B2	110.00
C8B—C7—C9B	110.3 (3)	C7—C10A—H10A	109.00
C8B—C7—C10B	110.33 (19)	C7—C10A—H10B	109.00
N1—C7—C9B	106.32 (17)	H10A—C10A—H10B	108.00
O3A—C10A—C7	112.3 (6)	O3A—C10A—H10A	109.00
O3B—C10B—C7	116.0 (4)	O3A—C10A—H10B	109.00
C2—C1—H1	120.00	O3B—C10B—H10D	108.00
C6—C1—H1	120.00	H10C—C10B—H10D	107.00
C3—C2—H2	120.00	C7—C10B—H10C	108.00
C1—C2—H2	120.00	C7—C10B—H10D	108.00
C2—C3—H3	120.00	O3B—C10B—H10C	108.00
C4—C3—H3	120.00		
			
O1—S1—N1—C7	43.70 (14)	C2—C1—C6—C5	0.3 (3)
O2—S1—N1—C7	173.39 (12)	C2—C1—C6—S1	179.33 (19)
C6—S1—N1—C7	−73.17 (13)	C6—C1—C2—C3	−0.6 (4)
N1—S1—C6—C1	126.00 (15)	C1—C2—C3—C4	0.8 (4)
O1—S1—C6—C1	7.45 (17)	C2—C3—C4—C5	−0.5 (4)
O2—S1—C6—C1	−121.01 (15)	C3—C4—C5—C6	0.2 (3)
N1—S1—C6—C5	−54.93 (15)	C4—C5—C6—C1	0.0 (3)
O1—S1—C6—C5	−173.48 (13)	C4—C5—C6—S1	−179.10 (14)
O2—S1—C6—C5	58.06 (15)	N1—C7—C10B—O3B	−56.0 (6)
S1—N1—C7—C9B	159.68 (18)	C8B—C7—C10B—O3B	174.2 (6)
S1—N1—C7—C8B	35.1 (2)	C9B—C7—C10B—O3B	56.1 (6)
S1—N1—C7—C10B	−90.15 (16)		

Symmetry codes: (i) −*x*+2, −*y*+1, −*z*+1; (ii) −*x*+2, *y*+1/2, −*z*+3/2; (iii) *x*, −*y*+3/2, *z*−1/2; (iv) *x*, −*y*+1/2, *z*−1/2; (v) −*x*+2, *y*−1/2, −*z*+3/2; (vi) −*x*+1, *y*−1/2, −*z*+3/2; (vii) *x*, −*y*+1/2, *z*+1/2; (viii) −*x*+1, *y*+1/2, −*z*+3/2; (ix) −*x*+2, −*y*+1, −*z*+2; (x) *x*, *y*+1, *z*; (xi) *x*, *y*−1, *z*; (xii) *x*, −*y*+3/2, *z*+1/2.

### Hydrogen-bond geometry (Å, °)

**Table:**

*D*—H···*A*	*D*—H	H···*A*	*D*···*A*	*D*—H···*A*
N1—H1A···O3B^v^	0.86	2.14	2.840 (9)	139
O3B—H3B···O2^i^	0.82	2.02	2.777 (10)	152
C1—H1···O1	0.93	2.51	2.886 (3)	105
C9B—H9B1···O3B	0.96	2.54	2.881 (11)	101
C10B—H10D···O1^xii^	0.97	2.54	3.478 (3)	162

Symmetry codes: (v) −*x*+2, *y*−1/2, −*z*+3/2; (i) −*x*+2, −*y*+1, −*z*+1; (xii) *x*, −*y*+3/2, *z*+1/2.
